# Ethical aspects of hemophilia gene therapy: a qualitative interview study with stakeholders

**DOI:** 10.1016/j.rpth.2023.102237

**Published:** 2023-10-19

**Authors:** Lieke Baas, Karina Meijer, Mariëtte Driessens, Annelien L. Bredenoord, Rieke van der Graaf, SYMPHONY consortium@SYMPHONY_NL, M.H. Cnossen, S.H. Reitsma

**Affiliations:** 1 Department of Bioethics and Health Humanities, Julius Center for Health Sciences and Primary Care, University Medical Center Utrecht, Utrecht University, Utrecht, The Netherlands; 2 Department of Hematology, University Medical Center Groningen, University of Groningen, Groningen, The Netherlands; 3 Netherlands Hemophilia Patients Society (NVHP), Nijkerk, The Netherlands; 4 Erasmus School of Philosophy, Erasmus University Rotterdam, Rotterdam, The Netherlands

**Keywords:** ethics, freedom, gene therapy, hemophilia, qualitative research

## Abstract

**Background:**

There are great expectations for the potential role of gene therapy in the treatment of hemophilia. At the same time, developments in the field of hemophilia gene therapy have always raised ethical issues. It remains unknown how these ethical issues are perceived by stakeholders, particularly regarding the most recent developments in the field.

**Objectives:**

To obtain insight into stakeholders’ morally reasoned opinions on gene therapy for hemophilia.

**Methods:**

We conducted qualitative research with Dutch people with hemophilia (*n* = 13), parents of children with hemophilia (*n* = 5), physicians (*n* = 4), nurses (*n* = 3), a regulator (*n* = 1), and a representative from a pharmaceutical company (*n* = 1). We conducted semistructured interviews based on a topic list and reported the results according to the Consolidated Criteria for Reporting Qualitative Research guidelines.

**Results:**

We identified 3 main themes. The theme *freedom and independence* describes the hope people with hemophilia have of increasing their freedom through gene therapy, as well as concerns that gene therapy increases their dependence on their treatment center. The theme *trust and altruism* describes how people with hemophilia have a high level of trust in their physician and treatment center as well as in scientific research. As a result of this trust, they are willing to participate in research to help other people with hemophilia. The theme *incremental benefits* describes doubts respondents have about the added value of gene therapy compared to standard treatment.

**Conclusion:**

Stakeholders embrace the theoretical potential of gene therapy, while several people with hemophilia question the added value of the current gene transfer products for themselves.

## Essentials

• It is unknown how stakeholders perceive the ethical aspects hemophilia gene therapy.
• We conducted an interview study with Dutch stakeholders to identify ethically relevant themes.
• Freedom and independence, trust and altruism, and incremental benefits are important ethical themes.
• People with hemophilia hope to enhance their autonomy, but it is unclear if gene therapy will enable this.

## 1 Introduction

Since research on hemophilia gene therapy started, there have been great expectations of its potential for the treatment of hemophilia. If left untreated, the lack of clotting factor (F) VIII (hemophilia A) or FIX (hemophilia B) leads to spontaneous and trauma-induced bleeding into muscles and joints. As a result, people with hemophilia suffer from chronic pain and loss of function [1,2]. Currently, several treatment options are available, with several advantages and drawbacks. Prophylactic clotting factor replacement therapy prevents bleeds for most people with hemophilia. However, i.v. injections are experienced as burdensome and the product is expensive. The burden of the injections has been mitigated by the recent introduction of extended half-life products which lower the frequency of i.v. injections, and the nonreplacement therapy emicizumab, which requires s.c. instead of i.v. injections [1,3,4]. However, as these options still require regular injections and offer no prospect of a cure, there are great expectations for gene therapy. In 2022, the first gene therapy products for hemophilia A and B received conditional market authorization [5,6].

Throughout its development, gene therapy has raised ethical concerns. When the first clinical trials were starting, the high levels of uncertainty surrounding these trials raised concerns about the acceptability of the risks imposed by these studies and raised questions about which patients to include [7,8]. Recently, ethical concerns have been identified concerning whether gene therapy can fulfill the (great) expectations, its impact on psychosocial aspects, and its high costs [9].

As hemophilia gene therapy research continues and the technique is further developed, expectations of what gene therapy could achieve are increasing [10]. However, it is unknown what stakeholders of hemophilia gene therapy hope to achieve with gene therapy and how the ongoing progress in the field affects their views on the desirability of gene therapy. Therefore, we conducted a qualitative interview study to identify stakeholders’ morally reasoned opinions on the ethics of gene therapy for hemophilia.

## 2 Methods

Qualitative interviews are a valuable method to identify and better understand perspectives. Thereby, interviews can improve our understanding of ethical implications of gene therapy [11]. To validate our findings, we conducted an expert meeting after roughly two-thirds of the interviews had been conducted [12]. The study is reported in accordance with the Consolidated Criteria for Reporting Qualitative Research [13].

### 2.1 Participant selection and recruitment

We included a wide variety of Dutch stakeholders. As the goal of this qualitative study was not to compare the perspectives of different groups of stakeholders, but to obtain an overview of the scope of perspectives and opinions regarding ethical aspects of hemophilia gene therapy, we made use of purposive sampling [14]. Purposive sampling allows to ensure that specific types of cases become part of the final sample [14]. We used this strategy to include participants from a variety of backgrounds, thereby allowing for a broad range of perspectives. As part of this range of perspectives, we also included people who were cured of hemophilia through liver transplantation, as they have a unique experience of first living with hemophilia and later living without hemophilia. This group was included, as one of the promises of gene therapy is that it will provide a definitive cure, irrespective of whether current gene therapies can deliver on this promise. Because gene therapy is currently only under development for severe hemophilia, we did not include (parents of) people with mild or moderate hemophilia.

In total, we interviewed 27 people with hemophilia who participated in a gene therapy trial (*n* = 4), people with hemophilia who underwent liver transplantation (*n* = 3), other people with hemophilia (*n* = 6), parents of children with hemophilia (*n* = 5), physicians (*n* = 4), nurses (*n* = 3), an employee from a pharmaceutical company (*n* = 1), and a regulator (*n* = 1). One of the included physicians and one of the included nurses specialized in hemophilia care for children, and the others in hemophilia care for adults. We included participants affiliated with all 6 hemophilia treatment centers in The Netherlands. Characteristics of the respondents can be found in Table 1.

**Table 1:** Respondent characteristics.

Role	*N*
Person with hemophilia A (no gene therapy trial participation)	6
Person with hemophilia B (no gene therapy trial participation)	-
Parent of child with hemophilia A	4
Parent of child with hemophilia B	1
Trial participant with hemophilia A	1
Trial participant with hemophilia B	3
Person who no longer has hemophilia after liver transplantation	3
Physician (involved in gene therapy trial)	2
Physician (not involved in gene therapy trial)	2
Nurse (involved in gene therapy trial)	1
Nurse (not involved in gene therapy trial)	2
Regulator	1
Employee pharmaceutical company	1

Potential participants were approached in various ways. We targeted people with hemophilia and parents of children with hemophilia through an advertisement in the newsletter and social media of the Dutch hemophilia patients’ society (Netherlands Hemophilia Patients Society [NVHP]), as well as through their health care providers. Most professionals were invited directly by one of the authors (L.B. or K.M.) and nurses were also invited during a meeting of the nurses’ society. Furthermore, both people with hemophilia and professionals were recruited through word of mouth. Recruitment was completed when saturation was reached regarding codes and meaning [15].

Before the interview, L.B. sent an email containing the information letter and consent form to the participants. Additionally, she had a telephone call with all people with hemophilia and parents before the interview. L.B. had already met some of the professionals prior to inviting them for an interview. Participants were informed that the aim of the study was to identify ethical aspects of gene therapy for hemophilia and that the study was part of the SYMPHONY consortium [16]. Participants who asked about the background of the interviewer were informed that L.B. (MSc MA) does not have a medical background, is trained in bioethics, and that this study is part of her PhD research.

One invited physician declined participation because they considered themselves not knowledgeable enough. Two people who are active in the patient community and who responded to the advertisement were invited to participate in the validation meeting instead because several respondents already had similar characteristics. Three physicians who were invited to participate in the validation meeting declined as they could not make the scheduled timeslot. Staff members of one treatment center were unwilling to invite their patients because they wanted to avoid overloading their patients with research participation requests.

### 2.2 Data collection

Semistructured interviews based on a topic list were conducted by L.B. The topic list was designed based on the experiential knowledge of the research team and a literature study conducted by part of the research team [9]. The list was continuously evaluated and adapted as the interviews progressed, to allow for the inclusion of new topics in further interviews. Although several participants brought up the costs of gene therapy, we did not include this as a topic, because the costs of gene therapy were unknown at the time of the study.

The interviews were conducted face-to-face at the participant’s home or workplace, in a public place, or digitally via Microsoft Teams, depending on COVID-19 restrictions and the participant’s preferences. The interviews were audio-recorded, transcribed verbatim, and pseudonymized. After the interview, L.B. took notes of impressions of the interview and relevant remarks made by the participant before or after the audio recorder was switched on.

During most interviews only the interviewer and participant were present. In some cases, a partner or child of the participant was in the room. Some interviews were briefly disrupted by another person, infant, or pet. During one digital interview with a person with hemophilia, colleagues of the participant were present in the background. As this participant was active in the patient community and frequently spoke in public about their experience of living with hemophilia, we do not expect that this impacted their responses. The interviews lasted between 30 and 108 minutes, with a median duration of 49 minutes.

### 2.3 Data analysis

The pseudonymized transcripts were analyzed thematically [17]. An initial list of codes was developed based on the topic list, familiarization with the data, and discussion within the research team. All interviews were coded by L.B. with NVivo 12 software. Ten interviews were double coded by an intern, after which the codes were compared and discussed to assert intercoder reliability [17]. The process started with open coding, during which memos were made. The developed codes and memos served as the basis for developing higher-order codes and themes in discussion with the entire research team. The meaning of individual text fragments was determined by interpreting them in the context of the entire interview. During the analysis, codes were constantly evaluated and adapted, and new codes were developed if necessary. We went back and forth between the different analysis steps to allow for constant comparison [18]. The analysis ultimately resulted in the development of interpretive higher-order themes.

As a member check, we conducted an expert meeting after 16 interviews to discuss the accuracy and interpretation of our results [12]. Participants in the expert meeting were people with hemophilia who are involved in the NVHP (*n* = 3), physicians (*n* = 3), and nurses (*n* = 2) affiliated with different hemophilia treatment centers.

## 3 Results

Our analysis identified 3 ethical themes: freedom and independence; trust and altruism; and incremental benefits. Each will be described in more detail below and will be illustrated with quotes from the interviews. Additional quotes belonging to each of the themes can be found in Table 2.

**Table 2:** Illustrative quotes.

Theme	Quote
Theme 1: Freedom and independence	
*Hopes of gene therapy*	“My own expectation is that gene therapy is going to normalize the life of a hemophilia patient. So, giving you a treatment that lasts a long time and that results in a constant factor 8 level, as a result of which you can just live a normal life” – R10
*Limits to increased independence*	“If you only inject yourself in special situations, for instance once a year, then you will lose that skill. And then the hospital will say: ‘we’re not letting you do this anymore’. And the hospital is probably inclined to say: ‘for that one time a year, we rather have you come here instead of fumbling on your own’. And that is a situation in which a lot of people will feel like they’re losing control” – R14
*Becoming hemophilia-free and identity*	“In the beginning [after the trial], I thought ‘it is Monday, I have to inject’. But no, I did not have to inject. And on Thursday morning I had the urge to collect all items to inject myself. But no, that was not necessary anymore. In the beginning that was a very strange experience of course. You have to get used to that” – R2
Theme 2: Trust and altruism	
*Trust in research*	“I have a lot of trust in the sense that I think that much progress can be achieved in the next few years in the development of new medication and treatments” – R17
*Trust in physician and treatment center*	“Some people are really trusting of their physician who says ‘Let’s do this [enroll in a trial], this might be something for you’. And then they say: ‘Sure, why not?’” – R12
*Altruism*	“When they called me to ask to participate in this study, I thought I’ll just participate. If I can assist in the further development of treatments for a person with hemophilia, I’ll do that” – R1
Theme 3: Incremental benefits	
*Comparison with standard of care*	“If you have to inject 180 times a year, then it really is an advantage if you get gene therapy and you don’t have to do that anymore. But if you have emicizumab, of which the burden for the patient really is a lot smaller, then this balance [between advantages and disadvantages] will be different” – R5
*Coping with declining effects*	“The factor levels keep decreasing. There is going to be a moment, I don’t know if it will be a year or two years or ten years, but there is going to be a moment in which I have to inject prophylaxis again. […] In the beginning, whenever I did a blood test, I immediately went to the website to check the results. And now I stopped doing that, because I noticed every time my factor levels went down I thought ‘shit’ […] and I started thinking about it a lot” – R20
*Fair distribution*	“I think the biggest ethical challenge will be how we are going to distribute this over the patients, because this is never going to be reimbursed for the entire population” – R4

### 3.1 Theme 1: Freedom and independence

#### 3.1.1 Hopes of gene therapy

Respondents hoped that gene therapy, or treatment for hemophilia more generally, could offer people with hemophilia the opportunity to live a more “normal,” carefree, and independent life. For many, this meant a life unrestricted by hemophilia.

Respondents described several hindrances that people with hemophilia encounter in daily life. As a result of their hemophilia, several respondents were not able to choose the career or hobby they desired. Instead, they had to be careful with physical activities. Most professionals and people with hemophilia mentioned that these hindrances were more severe for older people who did not receive prophylactic treatment in the past than for people who grew up receiving the available treatment. Many of the interviewed people with hemophilia expected that gene therapy would provide a sustained increase in clotting factor levels and thereby the certainty that they can undertake a wider range of physical activities without risking bleeds. Several respondents thought that this increase in clotting factor levels would lead to a decreased experience of pain and stiffness in joints that have been injured by previous bleeds.

Furthermore, the respondents described several hindrances that people with hemophilia encounter resulting from their prophylactic treatment. Some indicated that the regular injections were a constant reminder of having hemophilia, and expressed the hope that gene therapy would help them to think about their disease less frequently. Some respondents also indicated that they cannot structure their day as they wish, because of the need to schedule time for administering their prophylaxis. Several individuals mentioned difficulties when traveling such as having to bring medication on a holiday and having to choose holiday destinations in relative proximity to a treatment center, which made them feel restricted in their ability to travel abroad:

*“When you’re travelling, you have to bring your medication. And you have to prepare and you’re not free. You are never free. And that is…. I don’t want to say you’re a prisoner, but you are a prisoner of the system*” –R13

Furthermore, several respondents thought that gene therapy would facilitate handling other medical conditions or treatments. For example, some described experiences with health care providers who knew too little of hemophilia to provide proper care and expressed the hope that gene therapy will simplify a visit to the dentist or other medical procedures. Further, some respondents feared that when growing old, people with hemophilia might lose the ability to inject themselves with clotting factors, thereby becoming dependent on family members or nursing home staff, who might lack the necessary skills or time.

#### 3.1.2 Limits to independence

Although several respondents provided accounts of increased independence after gene therapy, either based on their own experiences or that of their patients, there were also several people with hemophilia who were concerned that the current results of gene therapy would make them more dependent on their treatment center. They expected gene therapy to only raise their factor levels to the range of mild hemophilia. They reasoned that with such results they would still need clotting factor replacement in case of an injury, while people with mild hemophilia are not allowed to have a supply of clotting factor at home:

*“The advantage of severe hemophilia compared to mild hemophilia is that we are allowed to have medicine in stock. People with mild hemophilia are not allowed to [have their own stock]. That is a handicap. Whenever you go on a holiday, you have to request extra medicine or find out if it is available abroad. I am not dependent on that; I always have it with me.”* –R15

Moreover, several physicians and nurses expressed the concern that people would become less aware of their hemophilia or consider themselves hemophilia-free after gene therapy. They provided several anecdotes of people who took more risks after a gene therapy trial, in a way that was, in their view, irresponsible. Several of them said that the behavior of people who changed from severe to mild hemophilia was similar to the conduct of people who have always had mild hemophilia. The risks that people took included both physical activities that could elicit a bleed, such as heavy gardening or sports, as well as not taking proper action when bleeding occurred:

*“The biggest issue with people with mild hemophilia is that they call too late in case of a bleed. And now you’re making these people a mild patient.”* –R14

#### 3.1.3 Becoming hemophilia-free and impacts on identity

Some respondents who had not received gene therapy hypothesized that they would need time to adjust to being hemophilia-free if they would ever choose it as a treatment. They thought it might feel “strange” to not rely on clotting factor injections anymore and to have fewer restraints in their lives. People who participated in a trial or received liver transplantation also described an initial feeling of disbelief after not requiring clotting factor injections anymore. Nevertheless, all the people we spoke to thought that becoming hemophilia-free was desirable.

We found no univocal indications that becoming hemophilia-free impacts a person’s identity. Some health care providers knew a patient who explicitly stated that after gene therapy they no longer felt as a patient, which they described as a change in identity. None of the trial participants and people who underwent liver transplantation experienced a change in their identity after “losing” hemophilia. One respondent said that they used to be chair of the patient society but had to give this up after receiving liver transplantation because they were no longer a person with hemophilia after this procedure. Later, this person characterized this remark as a joke and said that the decision to step down from this position fitted with his phase in life, which was retirement.

Many people with hemophilia said that they had never let hemophilia define them and that they still tried to do as many things as possible, despite having to take their hemophilia into account. In contrast, one person mentioned that he felt his hemophilia had prevented him from developing his masculinity, as he always had to be careful, and one nurse described some of their patients as “being their hemophilia.” Nonetheless, the people who described they never let their hemophilia define them spoke of the friendships they gained through the patient society and how their medical care could be a social activity. Additionally, they showed pride in their ability to inject themselves intravenously:

“*Since I was twelve, I inject myself. I can do it while drunk. I can do it on a ship. I can do it in a moving car.”* –R7

### 3.2 Theme 2: Trust and altruism

#### 3.2.1 Trust in research

From the interviews it became clear that respondents embrace the promises of gene therapy and trust the outcomes of research. Both people with hemophilia and health care professionals expressed that they were amazed by the potential of gene therapy and considered its goals admirable:

*“It is incredibly wonderful that these developments are here and that patients can be helped by […] such a miracle. It is obviously incredibly impressive that people can study for so long and learn that these things can be solved”* –R20

Several respondents looked back on the advances that have been made in the treatment of hemophilia during the last decades and considered this progress an example of what science can achieve. Based on this progress, several respondents were certain that the treatment options for hemophilia would continue to improve.

Moreover, some people with hemophilia explicitly indicated that they had a lot of knowledge about how clinical trials were conducted and said that they knew this was always done correctly. Many respondents reported that they were generally trusting of science, often contrasting themselves with people who oppose COVID-19 vaccinations.

#### 3.2.2 Trust in physician and treatment center

Both health care professionals and people with hemophilia mentioned that people with hemophilia have a lot of trust in their physician and their treatment centers:

*“A hemophilia patient, because it is a disease from cradle to grave, has a very important relationship with his treatment center, with his physician.”* –R24

Many respondents considered this trusting relationship to be very valuable, and a good starting point for discussing gene therapy. Several persons thought it is valuable that the regular treating physician can also be the person conducting the recruitment and informed consent process in a gene therapy trial. Parents and people with hemophilia argued that they would prefer to be informed about trials by their own physician because they knew their physician would offer them the best possible care. Several respondents also emphasized the importance of this relationship when making a shared decision on gene therapy as a treatment.

In contrast, some respondents were concerned that people with hemophilia might too readily trust and believe their physician and would not think critically when deciding to participate in a trial, simply because it was proposed by their physician. Several physicians feel a large responsibility because of the trust placed in them, which could sometimes feel like a burden.

#### 3.2.3 Altruism

Many respondents considered it important to participate in research because they had high hopes for the outcomes. Several of them also mentioned that this was a reason to participate in this interview study. Multiple trial participants also indicated that a reason to participate in a gene therapy trial was to advance science and help other people with hemophilia, expressing the hope that younger generations would have an easier life than they had themselves. One respondent also reflected on the value that the development of gene therapy for hemophilia may have for patients with other congenital disorders. Many respondents recognized that the current standard of care could not have been achieved without the participation of other people with hemophilia in trials.

### 3.3 Theme 3: Incremental benefits

#### 3.3.1 Comparison with standard of care

Although respondents embrace the potential of gene therapy, several people articulated that the current results of gene therapy were not what they hoped they would be. It was often mentioned that the standard of care is already good and that it is questionable whether gene therapy can trump this, given its risks and uncertainties. Several people with hemophilia and parents thought that gene therapy might be more valuable for others than for themselves or their child, such as for older, younger, or more physically active people.

Simultaneously, several respondents were concerned that people with hemophilia could not always distinguish between the promises of gene therapy in general, and the possibilities of the products that are currently being tested in trials or will be on the market soon:

*“What strikes me most is that when you approach persons with hemophilia to ask them to participate in [gene therapy] trials, you are mainly busy tempering their expectations”* –R23

Several respondents thought that the choice of gene therapy or another treatment would depend on individual preferences. Some considered that even if gene therapy becomes an approved treatment, it may not be desirable for every person with hemophilia. One nurse explicitly stated that they thought that gene therapy might not be suitable for everyone, depending on their personal situation. It was also argued that gene therapy might be less desirable for people with hemophilia A than for those with hemophilia B, as the introduction of emicizumab has improved the standard of care for hemophilia A, and that the results of trials for hemophilia A are regarded as less positive than the results of trials for hemophilia B.

Many respondents explained that they themselves or their patients were thinking about the optimal timing for gene therapy. Some respondents were convinced that more effective forms of gene therapy will become available and therefore preferred to wait for a variant that may benefit them more.

#### 3.3.2 Coping with declining effects

Several respondents suggested that people should receive psychosocial support throughout and after the process of gene therapy. They argued that this would be required to help people adjust to their hemophilia-free life, but also to help them cope in case the effects of gene therapy decline and they need to use prophylaxis again. One of the trial participants also described that he had experienced a decline in the effects of gene therapy, which was an unexpected worry for him.

#### 3.3.3 Fair distribution

Some respondents were concerned that when gene therapy reaches the market, there will be a higher demand than can be afforded. Therefore, they considered which patients would benefit the most. All respondents who discussed this topic argued that people with hemophilia who have breakthrough bleeds while using prophylaxis or who had difficulties with injections would benefit most from gene therapy. Respondents disagreed about whether nonadherence to prophylaxis would be a fair criterion. Some physicians argued that they would like for a central body to decide on how gene therapy should be distributed, thereby taking away the decision from physicians and treatment centers.

## 4 Discussion

This study provides insight into stakeholders’ morally reasoned opinions on several ethical aspects of gene therapy for hemophilia. Based on our analysis, we identified 3 main ethical themes: freedom and independence; trust and altruism; and incremental benefits. In the following section, we will relate our findings to broader discussions in the literature and highlight topics that are underexplored. Finally, we will list the strengths and limitations of this study and provided recommendations for further research.

### 4.1 Discrepancy between wishes and reality

Our results indicate that stakeholders have several hopes for gene therapy that may be unachievable. People with hemophilia hope to increase their freedom, but many fear that they will become more dependent after gene therapy. Many respondents hope that they will be able to forget about their hemophilia after gene therapy, but health care providers are concerned about the potential consequences when people pay insufficient attention to their hemophilia. Lastly, many people admire the overall goals of gene therapy but question the added value of gene therapy products that are currently in advanced stages of development.

A literature study previously conducted by a part of our research team raised questions about gene therapy’s potential to live up to the expectations of a cure. It also argued that the phrase “gene therapy” is in fact used to refer to a range of different techniques, including approaches that are currently not beyond preclinical research stages, such as gene editing [9]. Therefore, it is plausible that this discrepancy between wishes and perceived reality reflects interview participants using the term gene therapy to refer to different things: either the overall program of gene therapy or specific adeno-associated virus-mediated gene transfer methods that will shortly enter the clinic. This could explain how they simultaneously embrace the promises of gene therapy but would forego current products.

This may also explain why other studies found that most of their participants would be interested in having gene therapy for themselves or their children, while only roughly 30% of these participants considered themselves knowledgeable about gene therapy [19,20]. It can be hypothesized that the participants in these studies reflected on a more general notion of gene therapy, whereas several participants in our study had knowledge of trial results.

Several authors have argued for the importance of educating people with hemophilia about gene therapy, to allow them to make an informed decision [20,21]. We would add that for such education to be most effective, it would have to differentiate clearly between different forms of gene therapy and their potential, rather than educating people about a general idea of gene therapy.

### 4.2 Increasing autonomy

This study identified freedom and independence as important outcomes for people with hemophilia. Another interview study found that “liberation” and “control” were important outcomes of gene therapy for people with hemophilia [22]. All these concepts are related to the value of personal autonomy, which refers to a state of self-governance and having the possibility to act free from controlling influences and limitations [23]. This suggests that finding ways to increase the autonomy of people with hemophilia is beneficial to their quality of life. Similarly, others have suggested that the concept of a “hemophilia-free mind” can be used to guide future hemophilia care [24].

Increasing autonomy does not solely have to be achieved through new treatments, and our results suggest that it also cannot be reached by adopting innovative therapies exclusively. This study shows that the extent to which gene therapy can achieve the desired goals partly depends on the social structures and policies in which the therapy is embedded, as well as the behaviors that the therapy elicits. Whether increased freedom is achieved depends on the rules that the treatment centers adopt and the frequency of follow-up visits required. Similarly, whether gene therapy effectively decreases the number of bleeds people suffer is dependent on the level of care and precaution they continue to take. Therefore, obtaining the most value from gene therapy will require embedding the treatment in practices of care that foster autonomy through other means as well.

### 4.3 Hemophilia identity

In contrast to earlier findings of (academic and journalistic) research [25,26], our study did not identify fear of impacts on personal identity as an important topic for people with hemophilia. Nevertheless, it was a topic that some respondents talked or joked about, indicating that it is a known phenomenon within the hemophilia community. Furthermore, many respondents told stories about how hemophilia had shaped their lives. This suggests that at least for some of the people affected, hemophilia has had a role in shaping their identity, a finding that is consistent with several other qualitative studies [27,28].

While conducting the interviews, it became clear that people use the term “identity” to refer to different things; for instance to what they called “identity politics,” their experience of being themselves, or their character. Within philosophical literature, identity can be conceptualized in different ways as well [29]. Previous research suggested that the fear of a change in identity experienced by some people with hemophilia results from the “burden of normality” [25]. The burden of normality describes the experience of patients who have difficulty adjusting to a symptom-free life after treatment, a phenomenon that has mainly been observed for patients with epilepsy or Parkinson disease who have been treated with deep brain stimulation [30,31]. Alternatively, although our results do not provide clear evidence for this, it can be hypothesized that the fear of a change in identity results from the feeling of no longer being part of a hemophilia community, comparable to how being part of a community is an important aspect of d/Deaf identity [32]. Several of our respondents indicated that after their liver transplantation, they became less active in the patient community, a community within which they had developed friendships, but none of them reported having experienced this as negative or impacting their identity.

### 4.4 Strengths, limitations, and recommendations for future research

Although our research design allowed the inclusion of participants with a variety of backgrounds, there were some aspects for which there was homogeneity among the respondents. First, although we included several people with hemophilia who would currently not consider gene therapy for themselves, there were no people who were generally opposed to gene therapy. Second, many respondents mentioned that they participated in this study because they considered it important to contribute to science or the further development of gene therapy. As a result, there might be a bias toward people with a relatively positive attitude toward genetic therapies and a high level of trust in science in our study. Third, although we included people with hemophilia B who participated in a trial and a parent of a child with hemophilia B, our sample did not include people with hemophilia B who did not receive gene therapy. It can be hypothesized that people with hemophilia B have a more positive attitude toward gene therapy than people with hemophilia A, as there is no subcutaneous treatment available for hemophilia B and recent research indicates that gene therapy expression is more stable and durable for hemophilia B than for hemophilia A [10]. However, this is something that needs to be explored in further research. Last, we did not incorporate data on race/ethnicity as a social determinant of health, as this information should in principle not be collected according to Dutch law. However, we made sure that participants varied with regard to other social determinants of health, such as age and place of residence.

Nevertheless, our research offers insight into a wide variety of stakeholders’ opinions on hemophilia gene therapy and identifies challenges that can occur when gene therapy becomes part of the standard treatment arsenal. The incorporation of a validation meeting allowed us to check our preliminary results for accuracy and adapt our topic list to incorporate more detailed questions on identity.

Our results provide several directions for future research. To begin, it would be valuable to find ways to foster autonomy in treatment to improve the quality of life for people with hemophilia. Furthermore, it will need to be investigated whether people with hemophilia experience identity issues when gene therapy enters the market, and if so, how they could be supported in the best way.

In addition, although we excluded the topic from the current study, the costs of gene therapy are an important topic for further research. As our results indicate, stakeholders have concerns about the fair distribution of gene therapy because of the expected high costs. Recent history indicates that such worries are not unreasonable. For instance, Bluebird Bio, which develops gene therapies for several disorders other than hemophilia, recently withdrew its licenses from the European market after not reaching an agreement over reimbursement with governments. Similarly, the gene therapy Libmeldy, for metachromatic leukodystrophy, will not be covered by Dutch health insurance because the costs are too high, making it inaccessible to patients [33]. This indicates that the pricing and payment structures of gene therapies might also limit their accessibility in high-income countries.

### 4.5 Conclusion

As gene therapy for hemophilia is developing rapidly and the first products are currently entering clinical care, it is important to explore the ethical issues that gene therapy raises. We have shown that freedom and independence, trust and altruism, and incremental benefits are important ethical considerations for stakeholders. Our study indicates that although people with hemophilia embrace the general promises of gene therapy, the products that shortly enter the market are not necessarily considered to be superior to the current standard of care. People with hemophilia wish to become more autonomous, which cannot solely be achieved through gene therapy. Furthermore, although becoming hemophilia-free has had an impact on the (social) lives of some people with hemophilia, our study does not indicate that people with hemophilia experience or fear a change in identity as a result of gene therapy. The insights obtained help to guide the further development of gene therapies and their introduction into society in a responsible manner.

## References

[1] Mancuso M.E., Mahlangu J.N., Pipe S.W. (2021). The changing treatment landscape in haemophilia: from standard half-life clotting factor concentrates to gene editing. Lancet.

[2] Mendell J.R., Al-Zaidy S.A., Rodino-Klapac L.R., Goodspeed K., Gray S.J., Kay C.N. (2021). Current clinical applications of in vivo gene therapy with AAVs. Mol Ther.

[3] Peters R., Harris T. (2018). Advances and innovations in haemophilia treatment. Nat Rev Drug Discov.

[4] Ozelo M.C., Yamaguti-Hayakawa G.G. (2022). Impact of novel hemophilia therapies around the world. Res Pract Thromb Haemost.

[5] European Medicines Agency (2022). First gene therapy to treat severe haemophilia A. European Medicines Agency. https://www.ema.europa.eu/en/news/first-gene-therapy-treat-severe-haemophilia.

[6] European Medicines Agency First gene therapy to treat haemophilia B. European Medicines Agency 2022. https://www.ema.europa.eu/en/news/first-gene-therapy-treat-haemophilia-b.

[7] Kimmelman J. (2008). Staunch protections: the ethics of haemophilia gene transfer research. Haemophilia.

[8] DiMichele D., Miller F.G., Fins J.J. (2003). Gene therapy ethics and haemophilia: an inevitable therapeutic future?. Haemophilia.

[9] Baas L., van der Graaf R., van Hoorn E.S., Bredenoord A.L., Meijer K. (2023). The ethics of gene therapy for hemophilia: a narrative review. J Thromb Haemost.

[10] Leebeek F.W.G., Miesbach W. (2021). Gene therapy for hemophilia: a review on clinical benefit, limitations, and remaining issues. Blood.

[11] Rehmann-Sutter C., Porz R., Scully J.L. (2012). How to relate the empirical to the normative toward a phenomenologically informed hermeneutic approach to bioethics. Camb Q Healthc Ethics.

[12] Birt L., Scott S., Cavers D., Campbell C., Walter F. (2016). Member checking: a tool to enhance trustworthiness or merely a nod to validation?. Qual Health Res.

[13] Tong A., Sainsbury P., Craig J. (2007). Consolidated criteria for reporting qualitatibe research (COREQ): a 32-item checklist for interviews and focus groups. Int J Qual Health Care.

[14] Campbell S., Greenwood M., Prior S., Shearer T., Walkem K., Young S. (2020). Purposive sampling: complex or simple? Research case examples. J Res Nurs.

[15] Hennink M.M., Kaiser B.N., Marconi V.C. (2016). Code Saturation versus meaning saturation: how many interviews are enough?. Qualitative Health Research.

[16] Cnossen M.H., van Moort I., Reitsma S.H., de Maat M.P.M., Schutgens R.E.G., Urbanus R.T. (2022). SYMPHONY consortium: orchestrating personalized treatment for patients with bleeding disorders. J Thromb Haemost.

[17] Boeije H. (2009).

[18] Boeije H. (2002). A purposeful approach to the constant comparative method in the analysis of qualitative interviews. Quality & Quantity.

[19] van Overbeeke E., Michelsen S., Hauber B., Peerlinck K., Hermans C., Lambert C. (2021). Patient perspectives regarding gene therapy in haemophilia: interviews from the PAVING study. Haemophilia.

[20] Khair K., Steadman L., Chaplin S., Holland M., Jenner K., Fletcher S. (2021). Parental perspectives on gene therapy for children with haemophilia: the Exigency study. Haemophilia.

[21] Woollard L., Gorman R., Rosenfelt D.J. (2021). Improving patient informed consent for haemophilia gene therapy: the case for change. Ther Adv Rare Dis.

[22] Fletcher S., Jenner K., Pembroke L., Holland M., Khair K. (2022). The experiences of people with haemophilia and their families of gene therapy in a clinical trial setting: regaining control, the Exigency study. Orphanet J Rare Dis.

[23] Beauchamp T.L., Ashcroft R.E., Dawson A., Draper H., McMillan J.R. (2007). Principles of health care ethics.

[24] Krumb E., Hermans C. (2021). Living with a “hemophilia-free mind” – The new ambition of hemophilia care?. Res Pract Thromb Haemost.

[25] Fletcher S., Jenner K., Holland M., Chaplin S., Khair K. (2021). An exploration of why men with severe haemophilia might not want gene therapy: the exigency study. Haemophilia.

[26] Zhang S. (2018). Why I Don’t Want to Cure My Hemophilia With Gene Therapy. The Atlantic. https://www.theatlantic.com/science/archive/2018/08/hemophilia-gene-therapy-cure-identity/540987/.

[27] Reinicke K., Søgaard I.S., Mentzler S. (2019). Masculinity challenges for men with severe hemophilia. Am J Mens Health.

[28] Dolatkhah R., Shabanloei R., Ebrahimi H., Ghasempour M. (2021). Content analysis of identity challenges in patients with haemophilia: a qualitative study. Nurs Open.

[29] Schechtman M. (2010). Philosophical reflections on narrative and deep brain stimulation. J Clin Ethics.

[30] Gilbert F. (2012). The burden of normality: From “chronically ill” to “symptom free”. New ethical challenges for deep brain stimulation postoperative treatment. J Med Ethics.

[31] Wilson S., Bladin P., Saling M. (2001). The “burden of normality”: concepts of adjustment after surgery for seizures. J Neurol Neurosurg Psychiatry.

[32] McIlroy G., Storbeck C. (2011). Development of deaf identity: an ethnographic study. J Deaf Stud Deaf Educ.

[33] Geneesmiddel Libmeldy niet in verzekerd pakket | Nieuwsbericht | Rijksoverheid.nl n.d. https://www.rijksoverheid.nl/actueel/nieuws/2023/04/12/geneesmiddel-libmeldy-niet-in-verzekerd-pakket.

